# Association Between Physical Activity, Sedentary Time, and Quality of Life in Patients with Chagas Disease During COVID-19 Pandemic in Brazil: A Cross-Sectional Study

**DOI:** 10.3390/ijerph22071137

**Published:** 2025-07-18

**Authors:** Isis Gabrielli Gomes Xavier, Patrícia Mello Andrade, Rodrigo de Lima Vitor, Tayná Cruz Barros, Whesley Tanor Silva, Luciana Fernandes Portela, Marcelo Teixeira de Holanda, Luiz Henrique Conde Sangenis, Gilberto Marcelo Sperandio da Silva, Flavia Mazzoli-Rocha, Fernanda de Souza Nogueira Sardinha Mendes, Andréa Rodrigues da Costa, Marcelo Carvalho Vieira, Daniela Palheiro Mendes de Almeida, Cláudia Maria Valete, Alejandro Marcel Hasslocher-Moreno, Henrique Silveira Costa, Vitor Barreto Paravidino, Tatiana Rehder Gonçalves, Roberto Magalhães Saraiva, Mauro Felippe Felix Mediano

**Affiliations:** 1 Evandro Chagas National Institute of Infectious Diseases, Oswaldo Cruz Foundation, Rio de Janeiro 21040-360, RJ, Brazil; isisgabrielli@yahoo.com.br (I.G.G.X.); patricia.farmaceutica.rj@gmail.com (P.M.A.); rodrigovitor.762@gmail.com (R.d.L.V.); taynacruz.barros@gmail.com (T.C.B.); whesleytanor@gmail.com (W.T.S.); luciana.portela@ini.fiocruz.br (L.F.P.); marcelo.holanda@ini.fiocruz.br (M.T.d.H.); luiz.sangenis@ini.fiocruz.br (L.H.C.S.); gilbertomarcelo@gmail.com (G.M.S.d.S.); flavia.mazzoli@ini.fiocruz.br (F.M.-R.); fernanda.sardinha@ini.fiocruz.br (F.d.S.N.S.M.); andrea.costa@ini.fiocruz.br (A.R.d.C.); danielapalheiro@gmail.com (D.P.M.d.A.); claudia.valete@ini.fiocruz.br (C.M.V.); alejandro.hasslocher@gmail.com (A.M.H.-M.); roberto.saraiva@ini.fiocruz.br (R.M.S.); 2 Cardiac Rehabilitation Service, Aloysio de Castro State Institute of Cardiology, Rio de Janeiro 20261-005, RJ, Brazil; vieiramc@yahoo.com.br; 3 Department of Otorhinolaryngology and Ophthalmology, Federal University of Rio de Janeiro, Rio de Janeiro 21941-590, RJ, Brazil; 4 Postgraduate course in Rehabilitation and Functional Performance, Physiotherapy Department, Federal University of Vales do Jequitinhonha and Mucuri (UFVJM), Diamantina 39100-000, MG, Brazil; henriquesilveira@yahoo.com.br; 5 Department of Epidemiology, Institute of Social Medicine, State University of Rio de Janeiro, Rio de Janeiro 20550-013, RJ, Brazil; vparavidino@gmail.com (V.B.P.); tatianarehder@gmail.com (T.R.G.); 6 National School of Public Health, Oswaldo Cruz Foundation, Rio de Janeiro 21040-360, RJ, Brazil; 7 Department of Research and Education, National Institute of Cardiology, Rio de Janeiro 22240-006, RJ, Brazil

**Keywords:** exercise, chronic diseases, health behavior, sedentary behavior

## Abstract

Background: COVID-19 led to social isolation, potentially reducing physical activity (PA), increasing sedentary time, and lowering quality of life (QoL). This study investigated the association between these factors in patients with Chagas disease (ChD) during the pandemic. Methods: This cross-sectional study included 187 patients with ChD. PA and sedentary time were assessed by the IPAQ-short and QoL by the WHOQOL-Bref. The relationship between PA levels and sedentary time with QoL were assessed using unadjusted and adjusted generalized linear models. Results: The highest tertile of total PA was positively associated with the psychological (Exp β = 1.11; 95% CI: 1.02–1.22) and environmental (Exp β = 1.12; 95% CI: 1.01–1.23) QoL domains. The intermediate (Exp β = 1.12; 95% CI: 1.01–1.25) and highest (Exp β = 1.14; 95% CI: 1.02–1.27) tertiles of moderate-to-vigorous PA were positively associated with the physical domain. Similarly, both the intermediate (Exp β = 1.11; 95% CI: 1.01–1.22) and highest (Exp β = 1.12; 95% CI: 1.01–1.21) tertiles of moderate-to-vigorous PA were positively associated with the psychological domain. The highest tertile of sedentary time was associated with a decrease in the physical domain (Exp β = 0.88; 95% CI: 0.79–0.98). Conclusions: Higher levels of total and moderate-to-vigorous PA were associated with better QoL, while greater sedentary time was associated with poorer QoL.

## 1. Introduction

COVID-19 is an infectious disease caused by the SARS-CoV-2 coronavirus, which is highly pathogenic and spreads very quickly, contributing to the high number of people infected in a short period of time [1,2]. While COVID-19 by itself poses numerous detrimental effects on physical and mental health, its impact may be even more severe for individuals already living with chronic conditions, such as Chagas disease (ChD) [3,4,5]. ChD is a neglected tropical disease caused by the protozoan Trypanosoma cruzi, which is endemic in 21 countries in the Americas, affecting around 6 to 8 million people worldwide, including approximately 3 million people in Brazil [6,7]. Approximately 70 million people in the Americas remain at high risk of ChD in areas of active transmission, and global migration has transformed ChD into a global issue [8]. During the COVID-19 pandemic, approximately one-third of all deaths among ChD patients followed at our clinical center were attributed to COVID-19, with the presence of comorbidities, especially those with three or more comorbidities, linked to worse prognoses [9].

Among the strategies implemented to mitigate the spread of COVID-19, social isolation was widely adopted to limit exposure to the SARS-CoV-2, a measure particularly important for individuals with chronic conditions such as ChD, who may be at increased risk of severe complications [10]. Previous studies demonstrated that adherence to social isolation measures was higher among individuals with chronic diseases compared to the general population [11,12]. However, this intervention can negatively affect lifestyles, potentially leading to decreased physical activity (PA) levels, increased sedentary behavior, and a decline in quality of life (QoL)—a concern that is particularly relevant for individuals with ChD, who, overall, exhibit lower levels of PA compared to the general Brazilian population [13,14,15]. In this scenario, a better understanding of the consequences generated by social isolation during the COVID-19 pandemic period in ChD patients is of paramount importance to facilitate the implementation of intervention strategies to improve the health of this population, particularly those involving PA, which have been demonstrated to promote important health benefits in this population [16,17,18,19]. To the best of our knowledge, no previous study has explored the relationship between PA levels and sedentary time with QoL in ChD patients during the COVID-19 pandemic. This is an important area of investigation, as PA levels and sedentary time are generally associated with QoL in numerous previous studies, including those involving both sick and healthy individuals [20,21,22]. In the context of ChD—a neglected chronic condition—these associations become even more relevant, particularly during the COVID-19 pandemic, when restrictions and social isolation may have disproportionately affected health behaviors and overall QoL. Therefore, this study aimed to assess the association between PA levels and sedentary time with QoL in individuals with ChD during the COVID-19 pandemic period. We hypothesized that individuals with higher levels of PA and lower sedentary time would report better QoL, in both physical and mental health domains.

## 2. Materials and Methods

### 2.1. Study Design and Population

This cross-sectional study was conducted between November 2020 and June 2021 (during COVID-19 pandemic period) and included individuals diagnosed with ChD through two simultaneous positive serological tests (enzyme-linked immunosorbent assay, chemiluminescence, or indirect immunofluorescence). Participants were from both sexes, aged ≥ 18 years, and were under routine clinical care at a reference center for tropical and infectious diseases in Rio de Janeiro, Brazil, which provides complementary exams and multidisciplinary care. Exclusion criteria were the presence of other infectious diseases, immunosuppressive conditions, or non-Chagasic heart disease at the time of data collection. This study followed the STROBE reporting guidelines [23].

### 2.2. Sample Size

The sample size calculation was performed to estimate the prevalence of PA during the COVID-19 pandemic in ChD patients, which was the primary objective of the previously published study using the same dataset [15]. The sample size was determined based on data from Puccinelli et al. (2021) [24], which reported a 76.5% prevalence of physically active individuals in the Brazilian population during the COVID-19 pandemic. Considering the total number of patients with ChD regularly followed at the center of care for patients with ChD (approximately 900 patients) in 2020, and considering a 95% confidence interval, a 6% accuracy estimate, and increasing the sample size by 10% to account for potential losses and refusals, a total of 175 participants was necessary for this study. Although the sample size was not specifically calculated for the regression analyses, it is considered sufficient for the models fitted in this study. Based on the commonly used rule of thumb recommending 15 observations per predictor variable in regression models [25] and considering one main exposure and 10 covariates (11 predictors total), a minimum of 165 participants would be sufficient to ensure adequate statistical power for multivariate modeling.

### 2.3. Study Procedures

Patients were invited to participate during their outpatient appointments and underwent study procedures on two occasions. On the first occasion, during their regular medical visits, participants provided informed consent, underwent anthropometric measurements, and had blood samples drawn. The second occasion involved administering socioeconomic and clinical questionnaires via telephone (telemedicine consultation) within one month after the initial visit, as well as collecting clinical information from patients’ medical records.

### 2.4. Physical Activity and Sedentary Time (Exposure)

PA levels were assessed using the short version of the International Physical Activity Questionnaire (IPAQ), which has been adapted and validated for the Brazilian population [26]. The IPAQ-short included six questions addressing the duration and frequency of walking, moderate, and vigorous activities over the previous seven days [27]. Energy expenditure for each intensity was calculated in metabolic equivalents of task (METs) as follows: walking (3.3 METs); moderate (4.0 METs); and vigorous (8.0 METs) activities [28]. The total PA volume, expressed in MET minutes per week, was determined by adding up the total weekly time spent on walking, moderate, and vigorous activities [29]. In addition, moderate-to-vigorous PA was calculated by adding up the METs from moderate and vigorous activities.

Sedentary time per day was also assessed by IPAQ-short and quantified as the weighted average of the total time spent sitting on weekdays and weekends over the last seven days.

### 2.5. Quality of Life (Outcome)

QoL was evaluated using the Brazilian version of the World Health Organization Quality of Life assessment short-form (WHOQOL-Bref), a validated 26-item questionnaire. The questionnaire includes two items on the overall QoL and 24 items covering four domains: physical, psychological, social relationships, and environment. Each domain was scored from 1 (none) to 5 (very much), with higher scores reflecting a more positive perception [30]. The total score ranges from 4 to 20 points, which was converted to a 0–100 scale. The Portuguese (Brazilian) version was validated by Fleck et al. (2000) [30] and demonstrated good internal consistency, as well as adequate discriminant and concurrent validity, in addition to good reliability. The WHOQOL-Bref questionnaire is widely accepted for assessing QoL across diverse populations and health conditions, including its use in studies involving patients with ChD [31,32].

### 2.6. Covariates

Sociodemographic and clinical variables were considered as covariates to characterize the study population and to address the potential confounding for the associations between PA, sedentary time, and QoL (Table S1). Race was self-reported as white, black, mulatto, yellow, and indigenous and recategorized into white and non-white (black, mixed, yellow, and indigenous) for data analysis. Marital status was self-declared by the participant as single, married/stable, divorced, and widowed. Schooling was categorized by years of formal study into <9 years, ≥9 to 12 years, and ≥12 years. Family income was calculated as the total income of all household members, including wages, pensions, and any other income at the time of the collection. Information on the number of rooms and residents per domicile was collected through direct questioning.

Data on comorbidities, including hypertension, diabetes, dyslipidemia, respiratory diseases, cancer, kidney disease, hepatic conditions, and other chronic illnesses, were retrieved from medical records. Anthropometric measurements were obtained following standard procedures [33], with obesity defined as a body mass index (BMI) ≥ 30 kg/m^2^, calculated as weight (kg) divided by height squared (m^2^) (WHO 2000). The clinical presentation of ChD—classified as indeterminate (positive serology without specific cardiac or digestive abnormalities), cardiac (presence of ChD-related cardiac abnormalities), or digestive (presence of ChD-related digestive abnormalities)—was determined using medical record data, which included clinical evaluations, electrocardiographic and echocardiographic assessments, and digestive examinations, in accordance with the guidelines of the Second Brazilian Consensus on Chagas Disease [34]. Immunoglobulin M (IgM) and immunoglobulin G (IgG) analyses for SARS-CoV-2 were performed through the chemiluminescence test (Abott) in serum samples of all participants, following the manufacturer’s protocol. Information on psychological conditions, including anxiety, depression, fear, and sadness, was collected through direct patient inquiries using the following questions: “Did you experience anxiety during quarantine?”; “Did you experience depression during quarantine?”; “Were you afraid during quarantine?”; and “Did you feel sad during quarantine?”.

### 2.7. Data Analysis

Descriptive statistics comprised means and standard deviations for continuous variables, and absolute frequency and percentages for categorical variables. The associations between PA levels (total, moderate-to-vigorous, and walking) and sedentary time with QoL domains (physical, psychological, social relationships, environment, and overall QoL) were initially evaluated using linear regression models. However, due to the asymmetric and heteroscedastic nature of the residuals, these associations were subsequently examined using generalized linear models (GLMs) with a gamma distribution and a logarithmic link function, which are more appropriate for modeling skewed, non-normally distributed continuous outcomes. The beta coefficients of GLMs were exponentiated (Exp β) to facilitate the interpretation of the results. The exponentiated beta coefficients (Exp β) from GLMs represent the proportional change in the outcome for each one-unit increase in a continuous exposure or change in category of a categorical variable: values equal to 1 indicate no change; values less than 1 indicate a percentage decrease; and values greater than 1 indicate a percentage increase in the outcome. The models were fitted unadjusted and adjusted for age, sex, race, schooling, comorbidities including hypertension, diabetes, dyslipidemia and obesity, and presence of cardiac and digestive forms.

Data management was carried out with the Research Electronic Data Capture (REDCap) web application and the data analysis was conducted using Stata 13.0 software. Statistical significance was set at *p* ≤ 0.05 for all analyses.

## 3. Results

Out of 350 screened patients, 187 consented and were included in the analysis. Table 1 describes the general characteristics of the patients included in the study population, overall and stratified by tertiles of PA. The mean age of participants was 61.1 (+11.6) years; most of them were women (62.0%), self-reported mixed race (50.8%), and had completed less than nine years of schooling (69.5%). Overall, those in the highest tertile of PA were younger, and less likely to present comorbidities (hypertension, diabetes, and dyslipidemia).

Table 2 presents the association between tertiles of total PA and QoL domains. In unadjusted models, participants in the highest tertile of total PA exhibited a 10% higher score in the psychological domain (Exp β = 1.10; 95% CI: 1.00–1.20) and an 11% higher score in the environmental domain (Exp β = 1.11; 95% CI: 1.01–1.22) compared to those in the lowest tertile. After adjusting for potential confounders, the highest tertile of total PA remained positively associated with an 11% increase in the psychological domain (Exp β = 1.11; 95% CI: 1.02–1.22) and a 12% increase in the environmental domain (Exp β = 1.12; 95% CI: 1.01–1.23) of QoL when compared to the lowest tertile.

Table 3 presents the associations between tertiles of moderate-to-vigorous PA and QoL domains. In the unadjusted models, participants in the intermediate and highest moderate-to-vigorous PA tertiles exhibited a 13% (Exp β = 1.13; 95% CI: 1.02–1.26) and 16% (Exp β = 1.16; 95% CI: 1.04–1.29) higher physical QoL score, respectively, compared to those in the lowest tertile. Additionally, both the intermediate (Exp β = 1.12; 95% CI: 1.03–1.23) and highest (Exp β = 1.12; 95% CI: 1.02–1.22) tertiles of moderate-to-vigorous PA showed a 12% higher psychological QoL score in comparison to the lowest tertile. After adjusting for potential confounders, the intermediate and highest of the moderate-to-vigorous PA tertiles remained positively associated with the physical QoL domain, with 12% (Exp β = 1.12; 95% CI: 1.01–1.25) and 14% (Exp β = 1.14; 95% CI: 1.02–1.27) increases, respectively, compared to the lowest tertile. Similarly, both the intermediate and highest tertiles of moderate-to-vigorous PA remained positively associated with the psychological QoL domain, with increases of 12% (Exp β = 1.11; 95% CI: 1.02–1.22) and 11% (Exp β = 1.12; 95% CI: 1.01–1.22), respectively.

Table 4 presents the association between tertiles of walking and the QoL domains. No significant associations were observed between the tertiles of walking and any of the QoL domains.

Table 5 presents the association between tertiles of sedentary time and the QoL domains. In the unadjusted analysis, the highest tertile of sedentary time was associated with a 12% decrease in the physical domain of QoL (Exp β = 0.88; 95% CI: 0.79–0.98) compared to the lowest tertile. After adjusting for potential confounders, the highest tertile of sedentary time remained associated with a 12% decrease in the physical domain of QoL (Exp β = 0.88; 95% CI: 0.79–0.98) compared to the lowest tertile. No significant associations were observed for the other domains.

## 4. Discussion

This study demonstrated that ChD patients engaging in higher levels of PA reported improved QoL across the physical, psychological, and environmental domains during the COVID-19 pandemic. Conversely, increased time spent on sedentary activities was associated with a decline in QoL within the physical domain.

In accordance with our findings, a cross-sectional observational study carried out by our research group before the COVID-19 pandemic (between 2014 and 2017) suggested that increased PA levels were positively associated with the perception of QoL in the physical domain of patients with ChD [31]. In the same vein, previous studies with cross-sectional data showed a consistently positive association between PA levels and QoL in the general population [22,35,36]. Similarly, a systematic review showed robust evidence, primarily from randomized controlled trials, that PA enhances QoL and well-being in middle-aged and older adults when compared to minimal or no-treatment control groups [37].

The potential relationship between PA and QoL can be explained by the impact that disability and physical limitations have on QoL in people with ChD, as demonstrated by a recent theoretical model developed specifically for this population. The authors describe a complex network of factors that influence QoL in this specific population. In particular, lower levels of PA may be associated with lower QoL scores due to the resulting inability to perform activities of daily living (ADLs and IADLs), leading to absence from work. This not only causes functional impairments but also compromises patients’ autonomy, leading to other repercussions such as emotional issues, social isolation, and financial problems [38]. During the COVID-19 pandemic, both PA levels and QoL declined due to fear of contagion and government recommendations for social isolation, which may have, in turn, intensified the strength of the relationship between these variables, as observed in other populations [24,39].

Individuals in the highest tertile of total PA and in the intermediate and highest tertiles of moderate-to-vigorous PA had a better perception of QoL in the psychological domain. Studies have shown that increased PA levels have numerous benefits for mental health [40,41]. This has also been observed in the context of the COVID-19 pandemic, and the mechanisms for these benefits include increased neurogenesis, reduced inflammatory and oxidative markers, and improved self-esteem [42]. Systematic reviews and meta-analyses conducted in the general population, aiming to provide an overview of the effects of PA interventions on QoL, have shown that the physical and psychological domains of QoL improve significantly compared to controls [43] in healthy individuals ranging from adolescents to older adults [44,45]. Another systematic review of 58 studies found a positive association between PA and QoL, particularly in the physical and mental health domains, with benefits for vitality and pain reduction [46]. A cohort study analyzed the relationship between different domains of PA and quality of life in Brazilian adults over two years concluded that increased PA was positively associated with some physical and mental aspects of QoL [20]. Therefore, higher PA levels seem to be a beneficial strategy for improving QoL and should be encouraged for individuals with ChD.

Few studies have investigated the relationship between PA levels and QoL in the environmental domain. In our study, the highest tertile of total PA was positively associated with the environmental QoL domain. More physically active individuals may perceive their environment more positively due to greater motivation to engage in leisure activities or increased use of active commuting, as a result of their higher fitness levels—factors that are all considered in the environmental domain of QoL in the WHOQOL-Bref. However, the cross-sectional design of this study makes it difficult to establish the direction of this association, since most previous studies in the literature have examined the opposite direction, suggesting that better environmental conditions are associated with higher levels of PA. The environment has a major influence on people’s lifestyles and their ability to choose healthy habits [47], with some authors pointing out that higher levels of PA are found among individuals who have enough free time for leisure or access to the necessary resources and facilities for PA [48]. Moreover, the previous literature has demonstrated a relationship between PA levels and several characteristics of the built environment, facilitating an increase in PA levels [49,50,51]. Therefore, individuals who live in better environments, with more leisure opportunities and more resources, are more likely to practice PA.

Another important finding is that increased sedentary time was associated with reduced QoL in the physical domain. A possible explanation for this association is that prolonged sedentary behavior may lead to physical deconditioning, muscle weakness, and lower energy levels, all of which can negatively impact an individual’s physical functioning and QoL [52,53]. The COVID-19 pandemic contributed to increased time spent on sedentary activities, as social isolation and related restrictions confined many work and study activities to the home environment, resulting in increased sedentary behavior among adults [54]. Additionally, a significant portion of the population spent more time engaged in sedentary activities, such as watching television and using mobile phones and computers for entertainment [55]. In other words, lockdown measures may have contributed to an increase in sedentary behavior worldwide [56,57], which may not only be associated with poorer QoL, as demonstrated in our study and population, but may also pose a risk for the development of new conditions that can further impact patients’ QoL [58].

This study has some limitations. Its cross-sectional design limits our ability to establish a causal relationship between PA levels, sedentary behavior, and QoL in individuals with ChD, as a bidirectional association is possible. The lack of data on participants’ adherence to social isolation measures during the COVID-19 pandemic should also be considered a limitation of the study. However, considering that social isolation was a widely recommended public health strategy in Brazil—particularly for individuals with chronic health conditions, who have been shown to adhere more strictly to these measures than those without such conditions—we treated the entire COVID-19 pandemic period as a context in which participants likely adopted, at least partially, some degree of social isolation [11,12] Besides that, the results should be interpreted with caution, as the study was carried out in a national reference center for the treatment of infectious diseases, where patients are regularly monitored. Therefore, these results could not be extrapolated to all individuals with ChD, considering that both PA practice and QoL can be influenced by the context of each individual. Conversely, to the best of our knowledge, this is the first study on the association between PA and QoL in individuals with ChD during the COVID-19 pandemic, which reinforces the novelty of our findings. Notably, some evaluations were conducted remotely via telemedicine, allowing for continued patient assessments during this period.

## 5. Conclusions

Individuals with ChD that present higher levels of PA had a better perception of QoL in the physical, psychological, and environmental domains. In addition, an increase in sedentary time was associated with a reduction in QoL in the physical domain in this group. Therefore, these findings have important implications for individuals with ChD, as they suggest the need for targeted interventions to promote PA and reduce sedentary behavior in this population. Further research, including longitudinal and interventional studies, is needed to clarify the causal relationships between PA, sedentary behavior, and QoL in individuals with ChD.

## Figures and Tables

**Table 1:** Characteristics of participants included in the study (overall and according to tertiles of total PA) [15].

Variables	Overall	Total PA (MET · min^−1^ · Week^−1^)			*p*-Value for Trend
	*n* = 187	Lowest (*n* = 63; 513.3)	Intermediate (*n* = 62; 2025.7)	Highest (*n* = 62; 5378.9)	
Age (years)	61.1 (+11.6)	64.2 (+11.3)	59.6 (+10.3)	59.3 (+12.6)	0.02
Women	62.0% (116)	60.3% (38)	56.5% (35)	69.4% (43)	0.30
Race					
White	23.0% (43)	28.6% (18)	25.8% (16)	14.5% (9)	0.09
Black	29.8% (37)	15.9% (10)	24.2% (15)	19.4% (12)	
Mixed	50.8% (95)	52.4% (33)	45.2% (28)	54.8% (34)	
Others	6.4% (12)	3.2% (2)	4.8% (3)	11.3% (7)	
Married/stable union vs. others	52.9% (99)	49.2% (31)	61.3% (38)	48.4% (30)	0.93
Schooling (years)					
<9	69.5% (130)	69.8% (44)	70.9% (44)	67.7% (42)	0.69
≥9–12	13.9% (26)	9.5% (6)	17.7% (11)	14.5% (9)	
≥12	16.6% (31)	20.6% (13)	11.3% (7)	17.7% (11)	
Rooms per domicile	4.63 (+1.40)	4.73 (+1.62)	4.63 (+1.32)	4.52 (+1.22)	0.39
Residents per domicile	1.71 (+1.53)	1.86 (+1.59)	1.63 (+1.43)	1.65 (+1.57)	0.44
Up to 1 minimum wage per capita income (vs. above)	54.0% (101)	66.7% (42)	45.2% (28)	50.0% (31)	0.06
BMI (kg/m^2^)	28.2 (+ 5.11)	28.9 (+ 5.1)	27.2 (+4.8)	28.4 (+5.3)	0.57
Indeterminate form	32.6% (61)	25.4% (16)	35.5% (22)	37.1% (23)	0.16
Cardiac form	63.6% (119)	71.4% (45)	61.3% (38)	58.1% (36)	0.12
Digestive form	13.4% (25)	14.3% (9)	11.3% (7)	14.5% (9)	0.97
COVID-19 antibodies					
IgM					
Positive	11.2% (21)	11.1% (7)	9.7% (6)	12.9% (8)	0.75
Negative	88.8% (166)	88.9% (56)	90.3% (56)	87.1% (54)	
IgG					
Positive	18.7% (35)	15.9% (10)	16.1% (10)	24.2% (15)	0.24
Negative	81.3% (152)	84.1% (53)	83.9% (52)	75.8% (47)	
Comorbidities					
Hypertension	70.0% (131)	85.7% (54)	62.9% (39)	61.3% (38)	0.003
Diabetes	21.4% (40)	28.6% (18)	22.6% (14)	12.9% (8)	0.04
Dyslipidemia	51.9% (97)	61.9% (39)	50.0% (31)	43.6% (27)	0.04
Obesity	29.9% (56)	30.2% (19)	29.0% (18)	30.6% (19)	0.95
Respiratory disease	14.9% (28)	9.5% (6)	12.9% (8)	22.6% (14)	0.05
Non-chagasic heart disease	3.2% (6)	1.6% (1)	3.2% (2)	4.8% (3)	0.31
Other chronic diseases	14.9% (28)	15.9% (10)	16.1% (10)	12.9% (8)	0.64
Self-reported anxiety during quarantine	47.1% (88)	42.9% (27)	48.4% (30)	50.0% (31)	0.42
Self-reported fear during quarantine	51.3% (96)	53.9% (34)	51.6% (32)	48.4% (30)	0.53
Self-reported sadness during quarantine	56.2% (105)	55.6% (35)	54.8% (34)	58.1% (36)	0.78
Self-reported depression during quarantine	26.2% (49)	30.2% (19)	30.6% (19)	17.7% (11)	0.12

Race: others (yellow and indigenous); Marital status: others (single, divorced, widowed); BMI: body mass index; IgM: Immunoglobulin M; IgG: Immunoglobulin G; Other chronic diseases: cancer, kidney disease, liver disease, another chronic disease; METs: metabolic equivalent of the task. Data are mean (±SD) or % (*n*).

**Table 2:** Association between the tertiles of total PA with QoL domains.

		Unadjusted			Adjusted *		
QoL Domains (WHOQOL-Bref)	Tertiles	Exponential (β)	95% CI	*p*-Value	Exponential (β)	95% CI	*p*-Value
Physical domain	Lowest	Reference					
	Intermediate	1.01	0.91–1.13	0.85	1.01	0.91–1.13	0.81
	Highest	1.11	0.99–1.23	0.07	1.11	0.99–1.24	0.06
Psychological domain	Lowest	Reference					
	Intermediate	1.09	0.99–1.19	0.07	1.09	0.99–1.19	0.08
	Highest	**1.10**	**1.00–1.20**	**0.04**	**1.11**	**1.02–1.22**	**0.02**
Social domain	Lowest	Reference					
	Intermediate	1.02	0.94–1.10	0.62	1.01	0.93–1.09	0.82
	Highest	1.06	0.98–1.14	0.17	1.06	0.98–1.15	0.17
Environment domain	Lowest	Reference					
	Intermediate	1.01	0.92–1.12	0.78	1.01	0.91–1.12	0.85
	Highest	**1.11**	**1.01–1.22**	**0.03**	**1.12**	**1.01–1.23**	**0.03**
General QoL	Lowest	Reference					
	Intermediate	0.99	0.89–1.12	0.94	0.98	0.87–1.09	0.69
	Highest	1.02	0.91–1.15	0.69	1.02	0.91–1.15	0.71

* Model adjusted by: age, sex, race, schooling, comorbidities (hypertension, diabetes, dyslipidemia and obesity), cardiac form, and digestive form. Estimates in bold are statistically significant.

**Table 3:** Association between the tertiles of moderate-to-vigorous with QoL domains.

		Unadjusted			Adjusted *		
QoL Domains (WHOQOL-Bref)	Tertiles	Exponential (β)	95% CI	*p*-Value	Exponential (β)	95% CI	*p*-Value
Physical domain	Lowest	Reference					
	Intermediate	**1.13**	**1.02–1.26**	**0.02**	**1.12**	**1.01–1.25**	**0.03**
	Highest	**1.16**	**1.04–1.29**	**0.008**	**1.14**	**1.02–1.27**	**0.02**
Psychological domain	Lowest	Reference					
	Intermediate	**1.12**	**1.03–1.23**	**0.01**	**1.12**	**1.02–1.22**	**0.02**
	Highest	**1.12**	**1.02–1.22**	**0.02**	**1.11**	**1.01–1.22**	**0.03**
Social domain	Lowest	Reference					
	Intermediate	1.04	0.97–1.12	0.29	1.03	0.96–1.12	0.42
	Highest	1.06	0.98–1.15	0.15	1.06	0.98–1.15	0.18
Environment domain	Lowest	Reference					
	Intermediate	1.01	0.92–1.11	0.79	1.01	0.91–1.11	0.92
	Highest	1.06	0.96–1.17	0.26	1.06	0.95–1.17	0.31
General QoL	Lowest	Reference					
	Intermediate	1.09	0.98–1.22	0.13	1.09	0.97–1.21	0.15
	Highest	0.98	0.88–1.10	0.77	0.97	0.87–1.09	0.66

* Model adjusted by: age, sex, race, schooling, comorbidities (hypertension, diabetes, dyslipidemia and obesity), cardiac form, and digestive form. Estimates in bold are statistically significative.

**Table 4:** Association between the tertiles of walking with QoL domains.

		Unadjusted			Adjusted *		
QoL Domains (WHOQOL-Bref)	Tertiles	Exponential (β)	95% CI	*p*-Value	Exponential (β)	95% CI	*p*-Value
Physical domain	Lowest	Reference					
	Intermediate	0.93	0.84–1.04	0.19	0.92	0.83–1.03	0.15
	Highest	1.02	0.91–1.14	0.75	1.02	0.91–1.14	0.72
Psychological domain	Lowest	Reference					
	Intermediate	0.99	0.91–1.09	0.89	0.98	0.89–1.08	0.74
	Highest	1.07	0.97–1.17	0.16	1.07	0.98–1.18	0.14
Social domain	Lowest	Reference					
	Intermediate	0.95	0.88–1.03	0.21	0.94	0.86–1.01	0.09
	Highest	1.02	0.94–1.10	0.65	1.01	0.93–1.09	0.77
Environment domain	Lowest	Reference					
	Intermediate	0.96	0.88–1.06	0.41	0.95	0.86–1.05	0.32
	Highest	1.09	0.99–1.21	0.07	1.09	0.99–1.20	0.09
General QoL	Lowest	Reference					
	Intermediate	0.94	0.85–1.06	0.31	0.92	0.82–1.04	0.17
	Highest	0.99	0.88–1.11	0.88	0.99	0.88–1.11	0.81

* Model adjusted by: age, sex, race, schooling, comorbidities (hypertension, diabetes, dyslipidemia and obesity), cardiac form, and digestive form.

**Table 5:** Association between the tertiles of sedentary activity time with QoL domains.

		Unadjusted			Adjusted *		
QoL Domains (WHOQOL-Bref)	Tertiles	Exponential (β)	95% CI	*p*-Value	Exponential (β)	95% CI	*p*-Value
Physical domain	Lowest	Reference					
	Intermediate	0.92	0.83–1.03	0.13	0.93	0.83–1.03	0.15
	Highest	**0.88**	**0.79–0.98**	**0.02**	**0.88**	**0.79–0.98**	**0.02**
Psychological domain	Lowest	Reference					
	Intermediate	1.00	0.92–1.09	0.93	1.01	0.92–1.10	0.92
	Highest	0.94	0.86–1.03	0.19	0.95	0.87–1.05	0.33
Social domain	Lowest	Reference					
	Intermediate	1.02	0.95–1.11	0.54	1.03	0.95–1.11	0.46
	Highest	0.99	0.91–1.07	0.80	1.01	0.93–1.09	0.85
Environment domain	Lowest	Reference					
	Intermediate	0.95	0.87–1.05	0.31	0.96	0.87–1.06	0.45
	Highest	0.91	0.82–1.00	0.05	0.92	0.83–1.02	0.11
General QoL	Lowest	Reference					
	Intermediate	0.97	0.87–1.09	0.63	0.97	0.86–1.09	0.59
	Highest	0.91	0.81–1.02	0.09	0.93	0.82–1.04	0.21

* Model adjusted by: age, sex, race, schooling, comorbidities (hypertension, diabetes, dyslipidemia and obesity), cardiac form, and digestive form. Estimates in bold are statistically significative.

## Data Availability

The data presented in this study are available upon request from the corresponding author. The data are not publicly available due to ethical restrictions and the need to protect the privacy and confidentiality of the study participants, as data sharing was not included in the ethics committee’s approval.

## Supplementary Materials

The following supporting information can be downloaded at: https://www.mdpi.com/article/10.3390/ijerph22071137/s1, Table S1: Sociodemographic variables and categorization used for analysis.

## Abbreviations

The following abbreviations are used in this manuscript:

PA	Physical activity
QoL	Quality of life
ChD	Chagas disease
IPAQ	International Physical Activity Questionnaire
WHOQOL	World Health Organization Quality of Life
SARS-CoV-2	Severe Acute Respiratory Syndrome Coronavirus 2
