# Supplementary material for: Association Between Physical Activity, Sedentary Time, and Quality of Life in Patients with Chagas Disease During COVID-19 Pandemic in Brazil: A Cross-Sectional Study
Source: Int J Environ Res Public Health. 2025 Jul 18;22(7):1137. doi: 10.3390/ijerph22071137 (PMC12294154; doi:10.3390/ijerph22071137)
Supplement: Supplementary file 1 [file ijerph-22-01137-s001.zip › ijerph-3713955-supplementary.pdf]

Table S1. Sociodemographic variables and categorization used for analysis

Variable	Original categories	Recategorization (for analysis)
Race/ethnicity	White, Black, Mulatto, Yellow, Indigenous	White / Non-white (Black, Mulatto, Yellow, Indigenous)
Marital status	Single, Married or in a stable union, Divorced, Widowed	Not recategorized
Schooling	Self-reported years of formal study	<9 years / ≥9 to 12 years / ≥12 years
Family income	Total household income from all sources (wages, pensions, other) at time of collection	Not categorized in original; used as continuous or categorized if needed
Household composition	Number of rooms and number of residents per domicile (self-reported)	Used to calculate people per room ratio (if applicable)
